# Specialty Grand Challenge: Diversity Matters in Healthcare Professions Education Research

**DOI:** 10.3389/fmed.2021.765443

**Published:** 2021-11-15

**Authors:** Lynn V. Monrouxe, Jacqueline G. Bloomfield

**Affiliations:** ^1^Faculty of Medicine and Health, School of Health Sciences, The University of Sydney, Sydney, NSW, Australia; ^2^Faculty of Medicine and Health, School of Nursing and Midwifery, The University of Sydney, Sydney, NSW, Australia

**Keywords:** education, diversity, translation, international, rigor

## Introduction

Diversity matters. Yet *matters of diversity* in healthcare professions education provide us with multiple challenges in our specialty. Healthcare professions education research considers *who* healthcare professionals are, *what* they learn, and *how* they learn and practice. Research into healthcare professions education therefore encompasses a diversity of topic areas across the continuum: from the moment a person decides to become a healthcare professional, throughout undergraduate education, postgraduate years and continuous professional development. Thus, it considers the holistic nature of learning and practice, including role modeling for the nascent professionals, the development of professionalism and professional identities, interprofessional learning and working, practicing relationship-centered care and healthcare professionals' well-being.

In our Specialty Grand Challenge article, we explore diversity challenges within healthcare professions education. In doing so, rather than seeking to simplify and reduce the challenges highlighted, we seek to illuminate them so that researchers in our field can work together to explore ways of overcoming these challenges, broadening our conversations and creating new avenues for understanding them.

## Diversity Challenges Across Healthcare Professional Groups

As healthcare professionals, we are a diverse mix. We comprise a range of professional groups, from doctors, nurses, midwives and dentists to newer groups (e.g., physician assistants, occupational therapists, physical therapists and genetic counselors). While all having different responsibilities and diverse spheres of practice in health promotion, disease prevention and healthcare service, there is a fair degree of skill-mix within and across us: made even more visible as medical care modernizes (1). Furthermore, we are connected through our moral ideology of caring (2). But despite similarities, professional stereotypes exist. Interestingly, these include demarcations around curing vs. caring, leaders vs. team workers, diagnostic vs. technical and across power differentials (3). Stereotypical caricatures across different healthcare professionals are often formed early, with reinforcement (via direct experience or societal images) leading to ingrained opinions around *the other* profession, resulting in potentially negative interprofessional behaviors. This can cause problems within workplace settings (3). Thus, one challenge we face is overcoming professional differences to develop positive workplace cultures for the benefit of healthcare professionals' well-being and patient care (4).

With professional roles and occupational boundaries being flexible and malleable, when working across these boundaries, cooperation can occur. Cooperation comes in many forms, including doctors deferring to nurses' experience and expertise, resulting in productive interprofessional relationships and enhanced patient outcomes (2, 5). Thus, research has shown how newly qualified doctors learn from nurses who have an important role in junior doctors' procedural skill development, alongside their professional socialization, as they navigate the complexities of hospital cultures and practices (6). Furthermore, experienced doctors also learn from nurses. But why stop there? Other healthcare professionals are also well-positioned to share their knowledge through cross disciplinary education. Developing a deeper understanding of the processes through which interprofessional practice and collaboration that occurs, and has the potential to occur, is essential for us to overcome a range of interprofessional challenges around sharing knowledge and expertise. So one of the challenges here is to break through the notion that individual healthcare professions are homogenous entities bound in structural and professional hierarchies, and to acknowledge “fluctuation in the flow and intensity of work” and the factors that break down jurisdictional boundaries during everyday practices (7). This is especially important during crisis situations such as the current pandemic (8).

## Diversity Challenges Within Healthcare Professional Groups

Other challenges arise from the issue of demographic diversity. Although, there is diversity of healthcare professionals in terms of race, ethnicity, class, age, cultural beliefs, practices and so on, demographic diversity within healthcare professional groups has yet to mirror societal diversity. Thus, within Western cultures, across a range of specialties in medicine, there continues to be an underrepresentation of national diversity for workforce recruitment and retention (9, 10). And it is not just medicine who lack demographic diversity. Consider the nursing profession. With its origins firmly embedded in domestic service, it largely remains a female-dominated field (11), with male nurses being a minority group representing <12% of the international nursing workforce with some exceptions: 23% in the Netherlands, 38% in Jordan and 50% in Mauritius (12). This underrepresentation of males, alongside stereotyping of the profession as being inherently female, may result in some males feeling unsupported, discriminated against and being recipients of lateral violence during nursing education and practice (13). Other research, however, suggests male nurses (especially white and heterosexual), have an advantage over females in the nursing workforce, with greater career and promotion opportunities (14). This double bind around issues of diversity, including sexuality, race and gender, is common across a range of issues within healthcare professions education.

Issues of demographic diversity also brings forth challenges of stereotyping and discrimination in healthcare practice. For example, when an ethnically homogenous workforce cares for a diversity of patients, problems can arise due to healthcare providers' biases, cultural misunderstandings and language/communication barriers. These include: over-diagnosis of certain diseases due to implicit associations with genetically disposed conditions (e.g., African-American and sickle cell anemia) *and* conditions with no genetic pre-disposition (e.g., African-American and obesity/drug abuse); gatekeeping behaviors due to implicit beliefs around ability to pay for care (despite being illegal practice); perceptions that certain tests (especially for chronic conditions) are unnecessary for temporary migrants; misunderstanding of the diversity in which healthcare and illness are understood across cultural groups; and patients' lack of confidence in healthcare professionals' treatment and recommendations (15–17). Furthermore, patients and clients are not the only ones who suffer negative outcomes due to this disparity. Healthcare professionals also report dissatisfaction, and even feelings of failure, within these interactions (18). While the promotion of diversity and equity within healthcare professional groups has been advocated (16, 19), this is not without its own challenges.

## Diversity Challenges in Learning Processes

There is also diversity of approaches to training across, and even within, the healthcare professions. Indeed, if we think exclusively about medical training at a macro level (e.g., curricula, policy, regulatory bodies) there are major differences in the pathways to becoming a doctor, alongside a plethora of disparate terminologies referring to various factors within this training period (20). For example, differences in curricula include graduate vs. direct entry access, the length of undergraduate training (from 4 to 8 years), expected outcomes and even what happens at the end of undergraduate training (e.g., directly entering residency, or internship and/or mandatory social/national service period) (20, 21). A recent exploration of medical curricula across 50 countries identified six distinct pathways to becoming a doctor with a great deal of variability within each in terms of general structures, learning processes, qualification degree and licensing (21). This international diversity facilitates the accommodation of national economic, political and social contexts (21). However, such disparity in undergraduate and postgraduate learning structures in medical education remains problematic. Not only for students wishing to undertake some of their training overseas, but also in terms of medical education research translation due to the lack of constructive alignment across the learning processes being studied. Furthermore, when we consider the potential transferability to other healthcare professional groups, this disparity becomes greater.

The lack of clear agreement on terminology within healthcare professional groups is yet another example of diversity. For example, in the United Kingdom (UK), medical students completing their undergraduate curricula move into what is known as the *Foundation Programme* and become, for 2 years, *Foundation Doctors* (22). Extending beyond the initial 2 years, this period is also known in the UK as *postgraduate medical education*. In other countries, however, *postgraduate medical education* specifically refers to the initial 2 years following an undergraduate degree with new graduates being called PGY1s/PGY2s (standing for *postgraduate year 1/2*) (23). But that is not all: postgraduate medical trainees during the same 2 year period across (and within) different countries are known variously as *junior doctors, medical officers, housemen, residents* and *interns* (24). Furthermore, the term *internship* is also occasionally used in other healthcare professional groups including pharmacy, physiotherapy, nursing and midwifery (25, 26). Even more confusing, the *internship* phase of education can occur during undergraduate as well as the postgraduate phases, dependent upon the healthcare professional group. This overlapping and disorganized nomenclature, whereby similar terms mean different things, and different terms mean the same thing, is not restricted to the naming of stages, but extends to a range of other key constructs in healthcare professions education. For example, recent research found 279 words and phrases across 440 research articles for groups or individuals referred to as *faculty*: defined as “*a person or group of people who engage in some sort of activity that is intended to impact another person or group's development”* (27). Including *doctor, physician, educator, teacher, preceptor* and *mentor*, these terms are mapped onto four key roles (in healthcare, education, academia and in relation to learners), with some degree of geographical variation. This is situation challenging for researchers and educators wishing to share best practice across healthcare professions, and even within professions internationally.

The ways in which different healthcare professional groups learn similar content is also diverse. Consider anatomy learning and the different pedagogies deployed, curricula content and outcomes across healthcare professional groups (28). A range of approaches including lectures, small group learning, living and radiological anatomy (e.g., body painting, peer learning), virtual (e.g., virtual/augmented realities) and learning with cadavers or *silent mentors* have been examined (29). Not all healthcare professional groups are equal in terms of what is available to them. Some students have no access to human cadavers; and with the modernization of healthcare professions education, fewer curricula hours are being devoted to laboratory gross anatomy learning. Subsequently, challenges arise for healthcare profession education researchers when evaluating the relative efficacy of different learning methods and ascertaining how we might best educate specific healthcare student groups for future practice. This issue is not confined solely to anatomy but spans across a range of skills and knowledge learning.

## Diversity Challenges of Research Approaches

Although healthcare professions education research is a social science (30), many in the field are primarily trained as practicing healthcare professionals with relatively little exposure to educational/social science research methods (31). Indeed, healthcare students are often taught that “science provides a single and true account of the world based on observation, which enables formulation of causal laws linking the observations in a logical, mathematically expressed order” (32). It is therefore unsurprising, that when undertaking any kind of research endeavor, the answer to the question of “what works” seems paramount (33). However, there is a growing body of researchers in the field originating from a social science or educational background, or who undertake higher degree research studies, that bring with them broader philosophical outlooks from which to ground their research. Subsequently, healthcare professions education research has expanded the range of philosophies from which it draws to advance the field. There is now a diversity of disciplinary and theoretical work (31), each with its own philosophy of science and their associated ontology, epistemology, methodology and axiology (34, 35).

At one end of the philosophical continuum researchers tend to consider a *problem* to be investigated by developing a measurable and testable hypothesis to explain what is happening. Studies are systematically designed, data is collected and statistically analyzed to determine whether to reject the null hypothesis. This positivist paradigm is associated with the *hypothetico-deductive model* of science in which a particular identifiable and measurable reality exists (33). The idea is to identify causality between measurable variables or correlation alongside a lack of confounding factors.

The other end of the philosophical continuum includes social constructionist thinking (34, 36). Here, knowing is shaped in and through language, differing through time and place. Furthermore, social constructionist researchers focus on what people are *doing* with their talk as they narrate their experiences. Ontologically, constructionism adopts an essentially relativist stance. Although both radical and moderate versions operate, from this general position there is no assumed *truth*; no objective view the world (36). Rather, subjectivity of what is *real* centers around discursive processes, including *who* is talking, to *whom* they are conversing, for *what purpose*, and so on. To know something is to attend to *what* is being said and *how* it is conveyed (5, 36). Here there are no causal links between constructs, no regular patterns or universal laws. Rather, individual insights of subjective experiences are privileged.

When we consider the diversity of research approaches along this philosophical continuum, researchers encounter many challenges. Challenges around which approach to adopt for the question or problem at hand; how their own ways of knowing [i.e., personal epistemologies (37)] may influence their chosen research genre; multi-professional teamworking challenges whereby different team members' personal epistemologies might be at differing points along the continuum; combining research approaches; and even publishing challenges whereby editorial preferences for certain research approaches may limit opportunities for publication in certain journals. Furthermore, this can be challenging not only for producers of healthcare professions education research, but also for consumers (38).

## The Grand Challenge: Research Dissemination and Impact

Knowledge dissemination is fundamental for advancing healthcare professions education, and needs to “take into account the message, source, audience and, channel” (61). Indeed, dissemination is a communication process. As with all communication processes, the culture in which it occurs shapes the norms and values through which messages are conveyed and received. Furthermore, although educational research has been occurring for decades within and across healthcare professional groups, some have a longer history, are more prolific, and the degree to which disciplinary communities value and understand the range of research approaches is varied (31, 39). Taken together, the disparate cultures that have historically existed across our healthcare professional groups, we consider that the dissemination of research across professional groups and its' subsequent uptake, and impact to be the grandest of challenges we all face.

Healthcare professional educators are avid publishers and readers. Attesting to this, is the extensive number of healthcare related journals that exist and which publish our research [albeit not exclusively: (40)]. Traditionally, single professional groups tend to publish research in profession-specific journals. As essential vehicles for the dissemination of cutting-edge research, scientific discoveries and new-found knowledge, each one focuses on topics of interest particular to each professional sector. Diversity of publication across professional groups is often limited to a few cross-cutting education journals. Subsequently research dissemination across healthcare professional groups has been arguably constrained. This not only limits the reach and scope of education-focused research, but also its application. Sharing what we know and what we discover with other disciplines is essential. This requires the creation of new spaces. Publishing research related to professional healthcare education from a diversity of perspectives, and in new ways and new places, will not only propagate a shared understanding of new evidence but also advance its translation to healthcare practice.

As we lead the development of this new and exciting section in Frontiers in Medicine, we therefore now challenge you to submit manuscripts that address the issues we have raised in this article. As we invite you to contribute, we urge you to consider your audience and rise to the greatest challenge of all: producing rigorous research, that is accessible and applicable to a cross-disciplinary and internationally diverse readership. We are confident that the collaborative efforts of healthcare professions educators and researchers from across the world will enable us all to both celebrate and harness our diversity; ensuring the provision of quality, evidence-based education and healthcare internationally.

## Author Contributions

LVM led on the intellectual content and structuring of the main sections and provided feedback and the final edits. JB significantly contributed to the intellectual content of the article, provided feedback, and approved the final version. All authors contributed to the inception of the article.

## Conflict of Interest

The authors declare that the research was conducted in the absence of any commercial or financial relationships that could be construed as a potential conflict of interest.

## Publisher's Note

All claims expressed in this article are solely those of the authors and do not necessarily represent those of their affiliated organizations, or those of the publisher, the editors and the reviewers. Any product that may be evaluated in this article, or claim that may be made by its manufacturer, is not guaranteed or endorsed by the publisher.
